# An improved catalogue of putative synaptic genes defined exclusively by temporal transcription profiles through an ensemble machine learning approach

**DOI:** 10.1186/s12864-019-6380-z

**Published:** 2019-12-23

**Authors:** Flavio Pazos Obregón, Martín Palazzo, Pablo Soto, Gustavo Guerberoff, Patricio Yankilevich, Rafael Cantera

**Affiliations:** 1 Neurodevelopmental Biology Department, Instituto de Investigaciones Biológicas Clemente Estable, Montevideo, Uruguay; 2 Instituto de Investigación en Biomedicina de Buenos Aires (IBioBA), CONICET - Partner Institute of the Max Planck Society, Buenos Aires, Argentina; 3 Instituto de Matemática y Estadística “Prof. Ing. Rafael Laguardia”, Facultad de Ingeniería, UDELAR, Montevideo, Uruguay

**Keywords:** Synaptic genes, Machine learning, Temporal transcription profiles, Gene function prediction, *Drosophila melanogaster*

## Abstract

**Background:**

Assembly and function of neuronal synapses require the coordinated expression of a yet undetermined set of genes. Previously, we had trained an ensemble machine learning model to assign a probability of having synaptic function to every protein-coding gene in *Drosophila melanogaster*. This approach resulted in the publication of a catalogue of 893 genes which we postulated to be very enriched in genes with a still undocumented synaptic function. Since then, the scientific community has experimentally identified 79 new synaptic genes. Here we use these new empirical data to evaluate our original prediction. We also implement a series of changes to the training scheme of our model and using the new data we demonstrate that this improves its predictive power. Finally, we added the new synaptic genes to the training set and trained a new model, obtaining a new, enhanced catalogue of putative synaptic genes.

**Results:**

The retrospective analysis demonstrate that our original catalogue was significantly enriched in new synaptic genes. When the changes to the training scheme were implemented using the original training set we obtained even higher enrichment. Finally, applying the new training scheme with a training set including the 79 new synaptic genes, resulted in an enhanced catalogue of putative synaptic genes. Here we present this new catalogue and announce that a regularly updated version will be available online at: http://synapticgenes.bnd.edu.uy

**Conclusions:**

We show that training an ensemble of machine learning classifiers solely with the whole-body temporal transcription profiles of known synaptic genes resulted in a catalogue with a significant enrichment in undiscovered synaptic genes. Using new empirical data provided by the scientific community, we validated our original approach, improved our model an obtained an arguably more precise prediction. This approach reduces the number of genes to be tested through hypothesis-driven experimentation and will facilitate our understanding of neuronal function.

**Availability:**

http://synapticgenes.bnd.edu.uy

## Background

The synapse, a specialized contact between neurons, is currently of fundamental importance for our understanding of learning, memory and other brain functions. Assembly and function of neuronal synapses require the coordinated expression of a yet undetermined set of genes, which for simplicity will be called here “synaptic genes”. There is a broad consensus that only a fraction of the total number of synaptic genes have been identified so far [1, 2]. Due to the evolutionary conservation among synaptic genes, the knowledge obtained from studies in model organisms is very relevant for other species, including humans [2, 3].

Since the biological roles of the vast majority of known amino acid sequences remain partly or completely unknown [4], computational prediction of gene function is an open research problem of much relevance. In recent years diverse methodologies have been assayed, with a strong prevalence of machine learning approaches. The top-performing algorithms, architectures and training schemes are function-specific and context-dependent [5]. In a previous study [6], we implemented an ensemble machine learning model that assigned a probability of being a “synaptic gene” to each protein-coding gene of *Drosophila melanogaster.* The features to infer the synaptic function were the whole-body transcription levels of all protein-coding genes at 24 developmental stages, published by the modENCODE project [7]. As far as we know, this is the only study that predicts gene function relying exclusively on temporal transcriptions profiles obtained through NGS technologies. After an exhaustive bibliographic review, a set of genes for which a function in synapse formation and/or maturation, and/or neurotransmission, and/or plasticity and/or maintenance had been experimentally demonstrated was selected as a positive example and included in the training set. Genes fulfilling any of two biological criteria defined ad hoc were selected as negatives examples [6] (See Methods and Additional file 1). Our model intersected the results of three learning algorithms: k-nearest neighbours (kNN) [8], Random Forest (RF) [9], Support Vector Machines (SVM) [10]. These algorithms had been chosen after obtaining similar results with these and other algorithms during an exploratory study and because they are widely used and among those with the best average performance when applied to biological data [11, 12]. The classification threshold of the algorithms was set to meet the expected number of unknown synaptic genes (estimated a priori) at that time. We obtained a catalogue that we postulated to be highly enriched in genes for which a synaptic function was yet to be discovered.

**Fig. 1:**
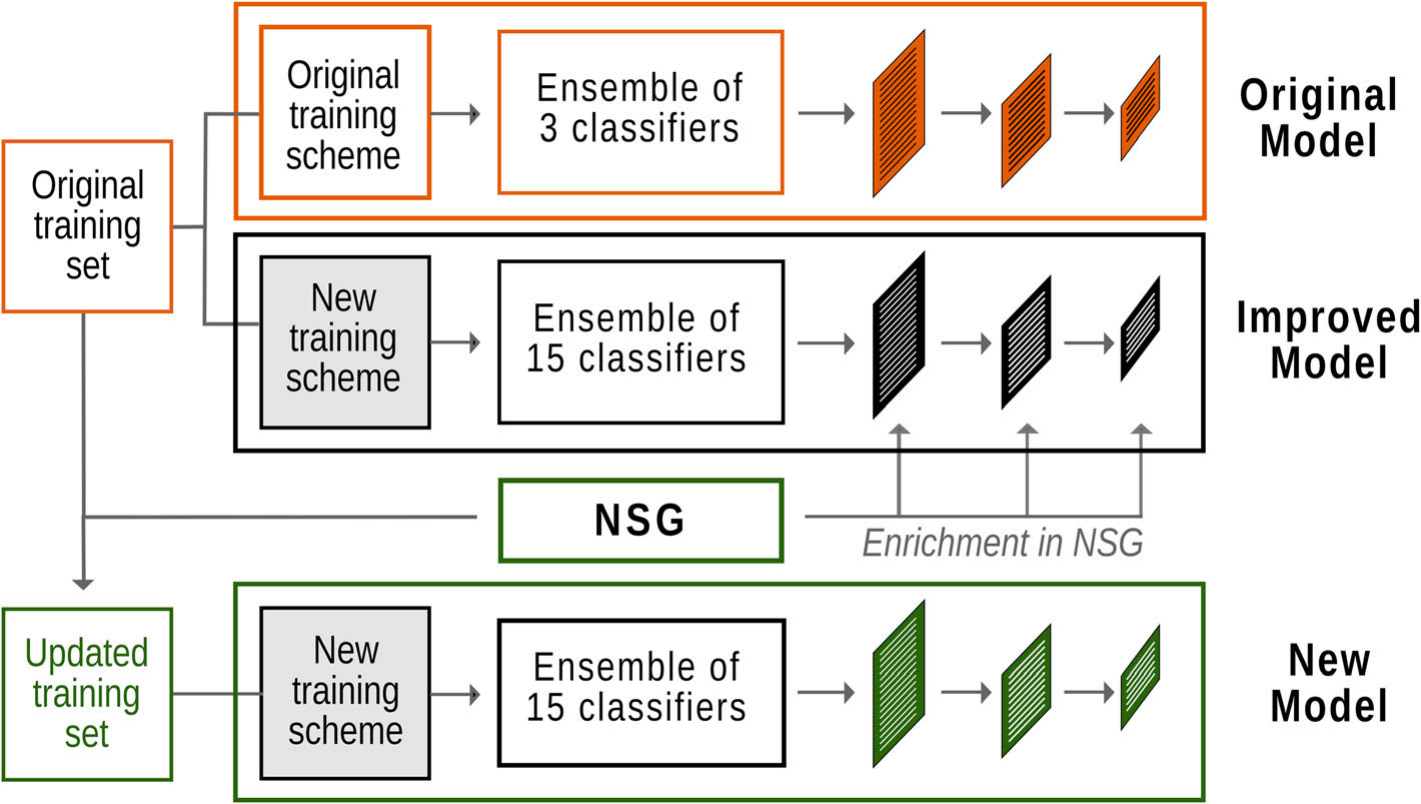
Scheme of the work-flow used to obtain the new catalogue of putative synaptic genes. First, we trained two models with the original training set, one with the original training scheme and one with the new training scheme (Fig. 2). Then we compared the enrichment in new synaptic genes (NSG, see Methods for definition of enrichment) of the catalogues resulting from each method. After testing that the new training scheme improved the prediction (Fig. 3), we incorporated the 79 NSG to the training set, trained a new model with the new training scheme and obtained a new catalogue of putative synaptic genes

Following the publication of that catalogue, scientists around the world have experimentally identified 79 new synaptic genes (NSG), giving us the opportunity to empirically evaluate the predictive power of the catalogue. Thereafter we tested a new training scheme and evaluated it by measuring the enrichment in NSG of the resulting catalogues. Briefly, the tested training scheme includes randomly sub-sampling the available labelled data to train a number of models and then ensemble those models in only one classifier (see Methods). This new training scheme is meant to alleviate a probable bias of our model due to a relatively small training set [13]. We found that the new training scheme resulted in a model producing a catalogue more enriched in NSG. Finally, we added the 79 NSG to the training set and trained a new model with the new training scheme. With this new model we obtained the new, enhanced catalogue of putative synaptic genes that we are publishing here. The whole procedure is schematized in Fig. 1. A monthly updated version of this catalogue will be available online at: http://synapticgenes.bnd.edu.uy.

## Results

### Evaluation of the original catalogue

Since the publication of our original catalogue up to the preparation of this manuscript, we identified 79 *Drosophila* genes that had gathered enough experimental evidence to be considered a synaptic gene according to our previous criteria [6]. Additional file 2 lists these genes along with the references supporting their synaptic functions. Roughly a third of these NSG (28 genes) were present in our original catalogue. A standard approach to evaluate the overrepresentation of certain feature (in this case, being a NSG) in a list of genes is to perform enrichment analysis (see Methods). Using in-house scripts and the hypergeometric distribution we calculated the enrichment in NSG of our original catalogue and its associated *p*-value. We found our original catalogue has an enrichment in NSG of 4.38 with a *p*-value < 10^− 10^ **(**Table 1**)**.

**Table 1:**
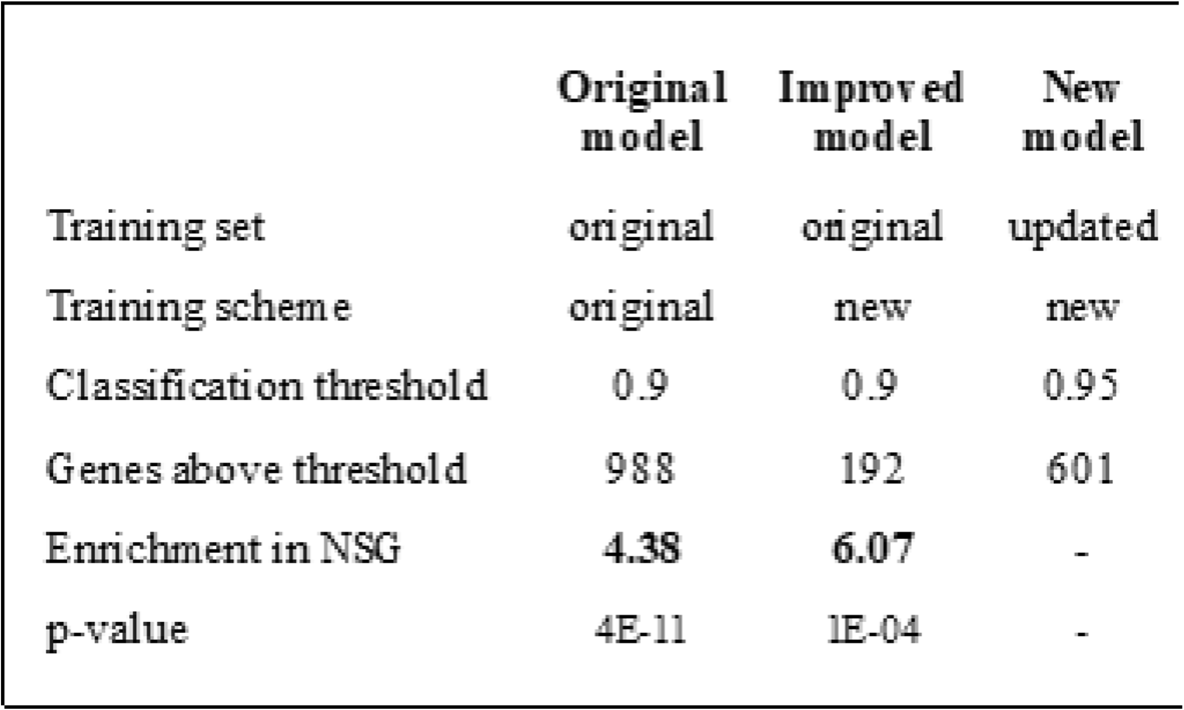
Evaluation of our original prediction. Comparison between the results of the original model, a model trained with the original training set but with the new training scheme and a model trained with the new training scheme and the updated training set. The enrichment in NSG found in the catalogue obtained with the new training scheme is 38% higher than that found in the catalogue obtained with the original training scheme even though both models were trained with the same set of genes. The training set for the new model includes the 79 NSG, thus the enrichment in NSG of the resulting catalogues cannot be defined

### Improved training scheme

**Fig. 2:**
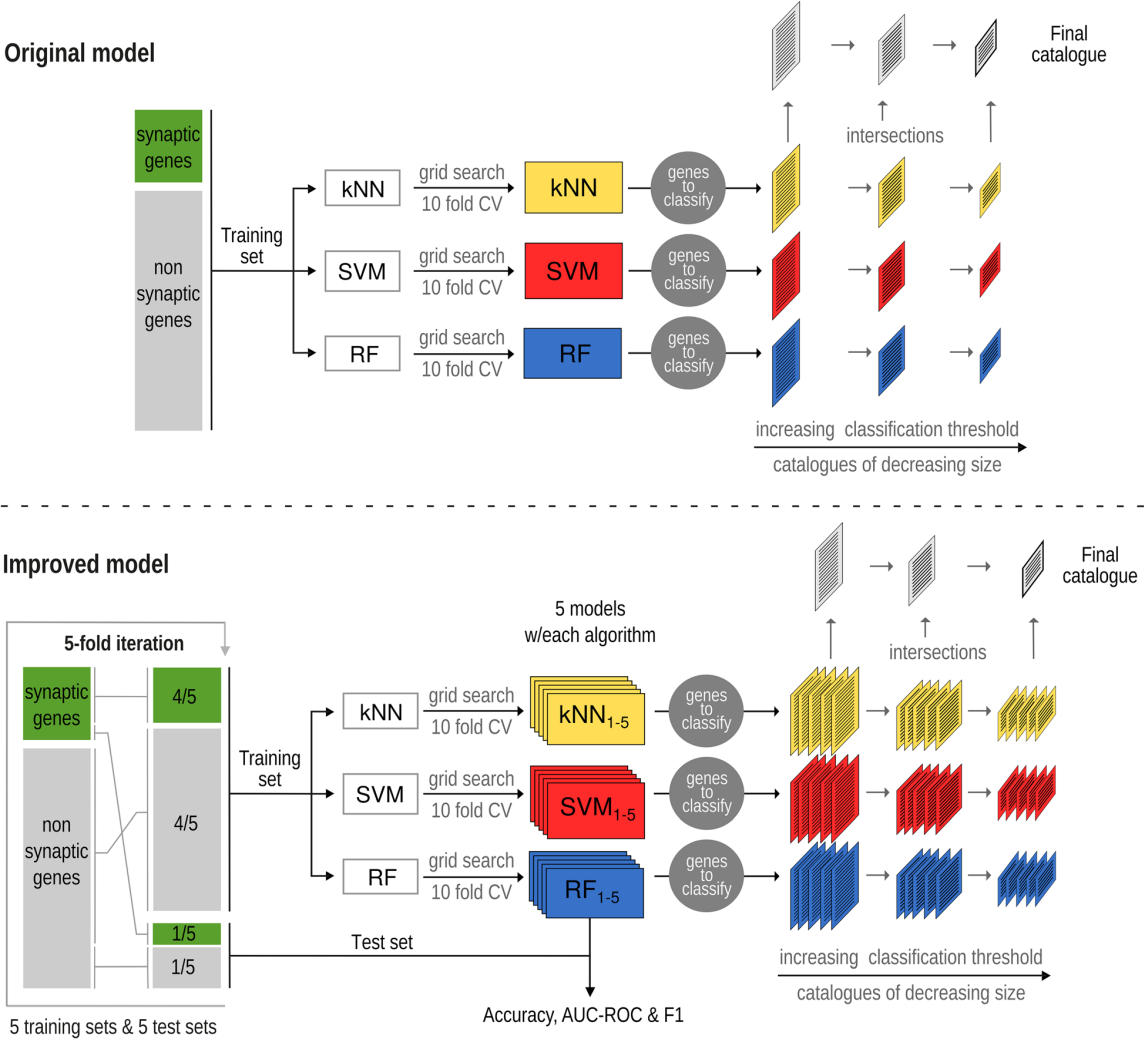
Comparison between the original and the improved models. Original model (above): with the whole training set we trained three algorithms: kNN, SVM and Random Forest. The hyper parameters of each classifier were set by exhaustive grid search combined with 10-fold cross validation over the training set. Finally, we increased the classification threshold of the classifiers and considered the intersection between the resulting catalogues. Improved model (below): first, we sub-sampled five times the original training set, leaving out each time a different fifth of the positive and negative examples. By this procedure we obtained five smaller, slightly different training sets. Using the positive and negative examples left out in each iteration, we created five test sets, used to independently evaluate each classifier. With each training set we trained three algorithms: kNN, SVM and Random Forest, thus obtaining 15 different classifiers. The hyper parameters of each classifier were set by exhaustive grid search combined with 10-fold cross validation over the training set. After training, we evaluated each classifier (accuracy, ROC and F1) using a different test set. Finally, we increased the classification threshold of the classifiers and considered the intersection between the resulting catalogues

The changes to the training scheme of our model tested here are detailed in the Methods section and schematized in Fig. 2.

**Fig. 3:**
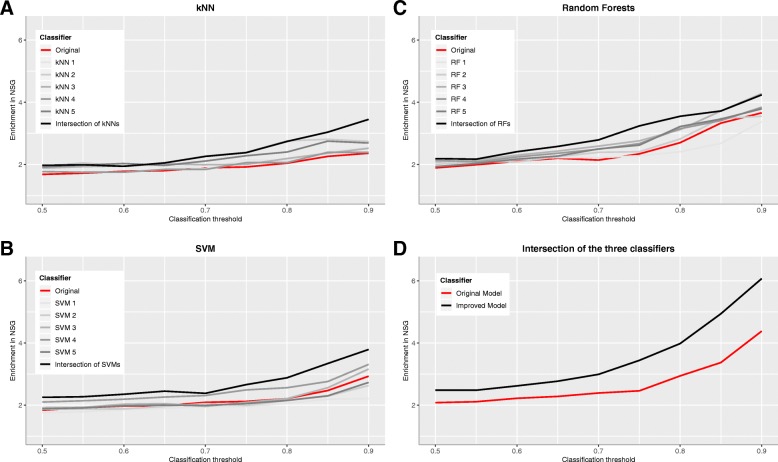
Comparison between the original and the improved model. Enrichment in NSG for an increasing classification threshold. **a**: kNN, **b**: SVM, **c**: Random Forest, **d**: Intersection of the three algorithms. For a definition of enrichment see Methods. In each panel, the red line represents the results of the original model, the gray lines represent the results of the models obtained after training the corresponding algorithm with each sub-sample of the original training set and the black line shows the results of the intersection of these last models

To test if these changes improved the predictive performance of our approach, we trained a model implementing these changes with the original training set and then compared the results with those of the original model. As shown in Table 1 and Fig. 3, the changes resulted in a better predictive power measured as enrichment in NSG. This improvement is also observed when we considered each classification algorithm separately (Fig. 3a-c). The performance of the intersection of the classifiers trained with the sub-samples of the training set was always better than that of the performance of the classifier trained with the full training set. By intersection of the classifiers for a given threshold we mean the set of genes that were assigned with a probability above the threshold by the 3 classifiers simultaneously.

### Evaluation of the new classifiers

After demonstrating that the proposed changes to the training scheme and ensemble rules would have resulted in a series of catalogues more enriched in NSG, we incorporated the 79 NSG to the training set and repeated the whole procedure described above, obtaining 15 new classifiers. Each of these classifiers was evaluated with an independent test set, which was used to calculate the accuracy, the F1 score and the area under the ROC curve (Fig.2 and Table 2). The obtained values were compared with those reported by other colleagues when training models to predict other biological functions [14–16].

**Table 2:**
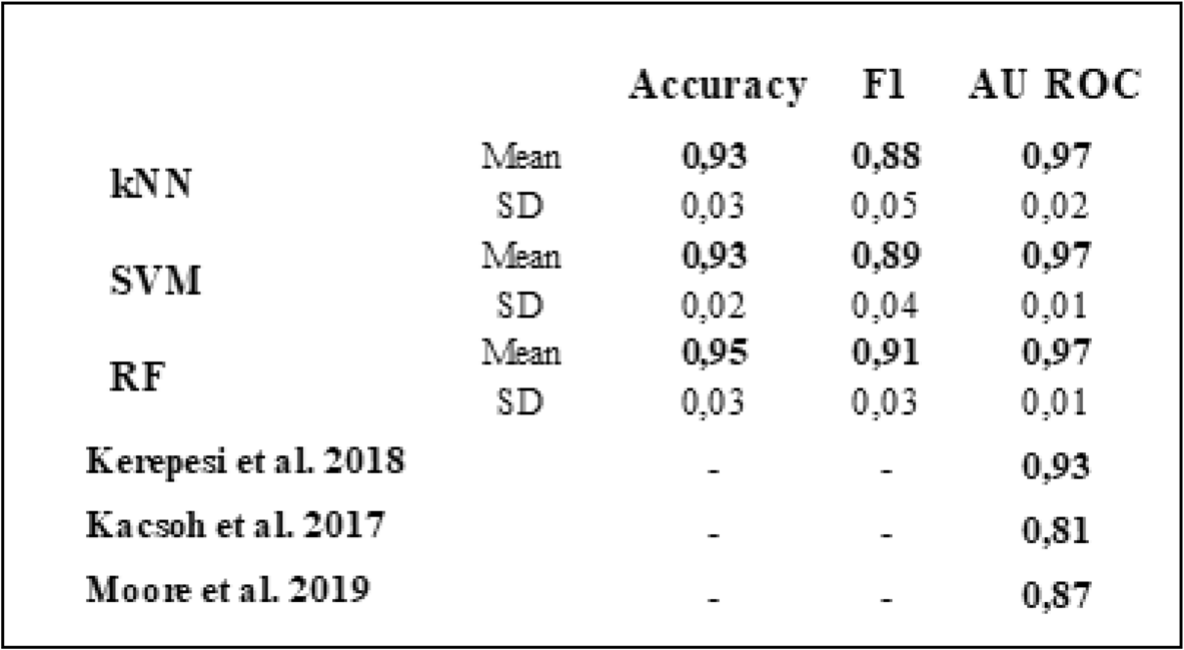
Evaluation of the 15 classifiers. Fifteen classifiers were obtained by training three algorithms with five different training sets. The performance of each classifier was evaluated using a test set conformed by genes that were not used during training. The table shows the mean and standard deviation of the accuracy, the F1 score and the area under the ROC curve of the five classifiers trained with each algorithm. The last three rows show the area under the ROC obtained by other colleagues when predicting other biological functions through machine learning

### A new catalogue of putative synaptic genes

We trained a new model incorporating the 79 NSG to the training set and the changes to the training scheme. In our original work only those genes assigned with a probability of being synaptic of at least 0.9 by the three classifiers were included in the final catalogue. This high classification threshold was set to obtain a catalogue of a given size. Now the threshold was set at 0.95 because we aimed to obtain a smaller catalogue since there are fewer unknown synaptic genes. The resulting catalogue had 601 genes.

### Enrichment of the new catalogue in synapse-related GO terms

To evaluate the quality of the new catalogue, we determined its enrichment in synapse-related GO terms. This could be done because we constructed our training set without taking into account Gene Ontology. We found that 83 of the 601 genes to which our 15 classifiers assigned a probability above 0.95 had some synapse-related GO annotation. To determine whether this is a significant enrichment, all the genes in our training set that have some synapse-related GO annotation must be removed from the background set. This analysis was performed with Gorilla [17] and the results are shown in Table 3. After excluding from the catalogue these 83 genes a final catalogue of 518 putative synaptic genes was obtained (Additional file 3).

**Table 3:**
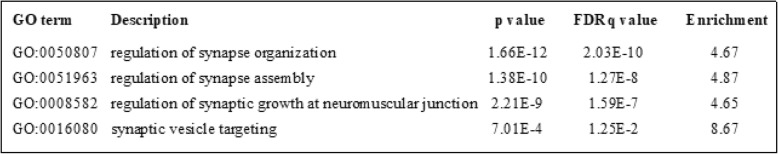
Enrichment of the new catalogue in synapse-related GO terms. First and second columns show the GO term identifier and its name. Third and fourth columns show the p-value and its correction for false discovery rate associated with the enrichment found, which is shown in the last column

### Regularly updated on-line catalogue

The model we are presenting here will be re-trained as new synaptic genes are identified. This will result in an updated catalogue that will be available here: http://synapticgenes.bnd.edu.uy. The updated list of synaptic genes used to train the model will be available at the same site.

## Discussion

An underlying rationale for our approach was that the transcription of genes of importance for neuronal synapses will probably increment very much during times of massive synapse assembly and will go down when synapses are massively degraded. A temporal correlation between changes in gene transcription and biological function has been reported for a variety of neuronal functions in *Drosophila* [18]. A catalogue obtained by training an ensemble machine-learning model that assigned each *Drosophila* protein-coding gene a probability of having synaptic function was published four years ago [6]. Of note, the model was based exclusively on a whole-body temporal transcriptome. It was hypothesized that the catalogue was enriched in genes of relevance for neuronal synapses which were still not recognized as such. Since the publication of the catalogue, 79 NSG were experimentally identified by others with a variety of experimental methods. This offered a great opportunity to test both the predictive power of our machine learning approach and to test changes to the training scheme that could improve the predictive power of our model. Here we found that our previous catalogue [6] was enriched in genes for which a synaptic function was experimentally identified by other colleagues between 2015 and 2019. We believe that this represents a good experimental validation of the predictive power of our machine learning approach and we conclude that it is thus possible to predict gene function using machine learning based exclusively on temporal transcription data.

Our original model assigned very low probabilities to some genes that were later proven to have synaptic functions. A possible explanation could be that our model cannot capture the entire diversity in expression profiles among the hundreds of genes required for assembly and function of neuronal synapses. It is suitable that any model exclusively trained with transcription profiles will fail to recognize some of the interesting genes, for several reasons and two of them will be considered in the following. Many genes have more than one biological function and are expressed at different levels in different organs or tissues. Hence, a machine learning approach applied to expression values obtained from total RNA samples from whole-organisms will probably fail to identify some of the genes of interest because of the composite nature of the sample. Moreover, the coordinated expression of hundreds of genes, during the two massive waves of synapse formation taking place during *Drosophila* development [6, 18] probably includes genes that encode activators or repressors, which will result in very different transcription profiles.

It is also worth noting that none of the 79 NSG belongs to the list of “non-synaptic genes” which had been used to train the algorithms. This provides unequivocal validation for the biological criteria used to select the negative examples of the training set and is consistent with our assumption that the genes of importance for neuronal communication are the same in both sexes.

It is important to note that even when the original model and its improved version were based on the same algorithms and were trained with the same set of genes, the enrichment in NSG found in the catalogue obtained with the improved model was 38% higher. This is interpreted as a clear demonstration that the new training scheme really improved the predictive performance of our approach. A possible explanation is that the tested training scheme alleviated a probable bias of our model due to a relatively small training set and increased its generalization capacity [19]. Since there are hundreds of synaptic genes to be discovered this is an important feature.

## Conclusions

We show here that a catalogue of *Drosophila* putative synaptic genes obtained by an ensemble machine learning model four years ago has a significant enrichment in genes whose synaptic function was discovered by others after its publication. This confirms that it is possible to predict gene function based on a temporal data-set of transcription values and a machine learning approach of the type presented here.

After testing the predictive power of our methods, we constructed a new catalogue of putative synaptic genes and make it available to the scientific community, firmly believing that this will facilitate the identification of genes important for the assembly and function of synapses, by means of gene silencing, mutant analysis, electrophysiology, neuroanatomy, behavioral assays and other traditional protocols, all of which will most likely lead to a better understanding of the function of the brain. The catalogue is available at: http://synapticgenes.bnd.edu.uy

## Methods

### Data

We used the developmental transcriptome of *Drosophila melanogaster* published by the MODENCODE Project [7]. In these data, each sample consisted of total polyAAA-RNA isolated from 30 whole bodies obtained at different time points along the organism life cycle. Originally the data set consisted of the transcript levels of 15,398 genes expressed as fragments per kilo base of exon per million fragments mapped (FPKM). We excluded 1756 genes that showed transcript levels above zero only during adult life and normalized each gene’s temporal series dividing it by its maximum value, thus obtaining for each gene a series of 24 values oscillating between 0 and 1. More details in [6].

### Evaluation of the predictive power of our original model

Using the same ad hoc definition for “synaptic gene” that was adopted in our previous work, we performed a bibliographic revision and identified 79 new synaptic genes defined as such by other scientists since the publication of our first catalogue [6]. Then we analyzed the enrichment of our original catalogue in these NSG using in-house scripts assuming a hyper-geometric distribution. If N is the number of genes in the background set, i.e. the set from which the analyzed list is extracted, B is the number of genes in the background set associated with the feature of interest, n is the number of genes in the analyzed list and b is the number of genes associated with the feature of interest in the analyzed list, the Enrichment is defined as ((b/n) / (B/N)).

### A new training scheme

To obtain our original catalogue we had trained three learning algorithms (k-NN, RF and SVM) with an unbalanced training set, in which there were many more negative than positive examples. A careful bibliographic revision was done to select genes for which the importance for neuronal synapses had been demonstrated with a variety of experimental approaches. In this way, 92 genes were selected as positive examples (Additional file 1). As negative examples, 397 genes were selected based on two biological criteria: genes that are not expressed at developmental stages when massive synapse formation takes place and genes with no expression during adult life in females or males, because available data indicate that the fundamental principles of structure and function of neuronal synapses are the same in both sexes.

With the aim of improving the predictive power of our model, here we propose a new training scheme, based on repetitive sub-sampling of the original training set to construct five smaller, slightly different training sets (**see** Fig. 1). To construct each of these training sets, four fifths of the original positive examples and four fifths of the original negative examples were randomly picked out. This procedure was repeated five times, leaving out a different fifth each time. Using the positive and negative examples left out when constructing each training set we defined a test set, used to independently calculate the accuracy, the AUC of the ROC and the F1 score of each classifier.

The hyper parameters of each classifier were set by grid search combined with 10-fold cross validation and its performance was evaluated by an independent test set. Each classifier assigned a different probability of being synaptic to each gene. To obtain our catalogues we considered, for each classification threshold, the intersection of the 15 results and then we took the mean probability assigned to each gene in the intersection.

All calculations were performed using Jupyter Notebooks and Sklearn [20].

## Supplementary information

**Additional file 1:** List of genes in the original training set (XLS 41 kb)

**Additional file 2:** New synaptic genes & references (XLS 21 kb)

**Additional file 3:** New catalogue of putative synaptic genes (XLS 39 kb)

## Data Availability

All data generated or analyzed during this study are included in this published article [and its supplementary information files].

## References

[1] Frank CA, Wang X, Collins CA, Rodal AA, Yuan Q, Verstreken P (2013). New approaches for studying synaptic development, function, and plasticity using Drosophila as a model system. J Neurosci.

[2] Laßek M, Weingarten J, Volknandt W (2015). The synaptic proteome. Cell Tissue Res.

[3] Burkhardt P (2015). The origin and evolution of synaptic proteins – choanoflagellates lead the way. J Exp Biol.

[4] UniProt CT (2018). UniProt: the universal protein knowledgebase. Nucleic Acids Res.

[5] Jiang Y, Oron TR, Clark WT, Bankapur AR, D’Andrea D, Lepore R (2016). An expanded evaluation of protein function prediction methods shows an improvement in accuracy. Genome Biol.

[6] Pazos Obregón F, Papalardo C, Castro S, Guerberoff G, Cantera R (2015). Putative synaptic genes defined from a Drosophila whole body developmental transcriptome by a machine learning approach. BMC Genomics.

[7] Graveley BR, Brooks AN, Carlson JW, Duff MO, Landolin JM, Yang L (2011). The developmental transcriptome of Drosophila melanogaster. Nature.

[8] Altman NS (1992). An introduction to kernel and nearest-neighbor nonparametric regression. Am Stat.

[9] Breiman Leo (2001). Machine Learning.

[10] Vapnik V (2000). The nature of statistical learning theory.

[11] Caruana R, Niculescu-Mizil A (2006). An empirical comparison of supervised learning algorithms.

[12] Fernández-Delgado M, Cernadas E, Barro S, Amorim D (2014). Do we need hundreds of classifiers to solve real world classification problems?. J Mach Learn Res.

[13] Dietterich TG (2000). Ensemble Methods in Machine Learning. Multiple Classifier Systems.

[14] Kacsoh BZ, Greene CS, Bosco G (2017). Machine Learning Analysis Identifies Drosophila Grunge/Atrophin as an Important Learning and Memory Gene Required for Memory Retention and Social Learning. G3 GenesGenomesGenetics.

[15] Kerepesi C, Daróczy B, Sturm Á, Vellai T, Benczúr A (2018). Prediction and characterization of human ageing-related proteins by using machine learning. Sci Rep.

[16] Moore BM, Wang P, Fan P, Leong B, Schenck CA, Lloyd JP (2019). Robust predictions of specialized metabolism genes through machine learning. Proc Natl Acad Sci.

[17] Eden E, Navon R, Steinfeld I, Lipson D, Yakhini Z. GOrilla: a tool for discovery and visualization of enriched GO terms in ranked gene lists. BMC Bioinformatics. 2009;10:48. 10.1186/1471-2105-10-48 PMC2644678 19192299

[18] Cantera R, Ferreiro MJ, Aransay AM, Barrio R (2014). Global gene expression shift during the transition from early neural development to late neuronal differentiation in Drosophila melanogaster. PLoS One.

[19] Hastie T, Tibshirani R, Friedman JH (2009). The elements of statistical learning data mining, inference, and prediction.

[20] Pedregosa F, Varoquaux G, Gramfort A, Michel V, Thirion B, Grisel O (2011). Scikit-learn: Machine Learning in Python. J Mach Learn Res.

